# Pathomechanisms of AERD—Recent Advances

**DOI:** 10.3389/falgy.2021.734733

**Published:** 2021-09-10

**Authors:** Annina Lyly, Tanya M. Laidlaw, Marie Lundberg

**Affiliations:** ^1^Department of Otorhinolaryngology – Head and Neck Surgery, Helsinki University Hospital, University of Helsinki, Helsinki, Finland; ^2^Inflammation Center, Skin and Allergy Hospital, Helsinki University Hospital, University of Helsinki, Helsinki, Finland; ^3^Department of Medicine, Division of Allergy and Clinical Immunology, Brigham and Women's Hospital and Harvard Medical School, Boston, MA, United States

**Keywords:** asthma, CRSwNP, samter's triad, N-ERD, aspirin-exacerbated respiratory disease, NSAID-exacerbated respiratory disease, nasal polyp, chronic rhinosinusitis

## Abstract

The pathomechanisms behind NSAID-exacerbated respiratory disease are complex and still largely unknown. They are presumed to involve genetic predisposition and environmental triggers that lead to dysregulation of fatty acid and lipid metabolism, altered cellular interactions involving transmetabolism, and continuous and chronic inflammation in the respiratory track. Here, we go through the recent advances on the topic and sum up the current understanding of the background of this illness that broadly effects the patients' lives.

## Introduction

AERD or aspirin/non-steroidal anti-inflammatory drug (NSAID)-exacerbated respiratory disease is an adult-onset triad characterized by asthma, eosinophilic chronic rhinosinusitis with nasal polyposis (CRSwNP), and respiratory reactions upon ingestion of COX-1 inhibitors such as NSAIDs or aspirin (ASA). Both the upper and lower respiratory symptoms are typically difficult-to-treat, and many patients suffer from frequent asthma exacerbations and require multiple endoscopic sinus surgeries despite good medical treatment including local and oral corticosteroids.

The acute inflammatory symptoms that occur within 30–180 min after ingestion of COX-1 inhibitors is a non-immunoglobulin (Ig)E-mediated hypersensitivity reaction that causes a release of multiple mast cell mediators, such as tryptase, cysteinyl leukotrienes (CysLTs), and prostaglandin D2 (PDG_2_) (1–3). The typical symptoms of reaction can include nasal congestion, rhinorrhea, sneezing, coughing, wheezing, and drop in lung function, though pruritic rash and gastrointestinal discomfort have also been noted.

### Arachidonic Acid Metabolism

**Figure 1:**
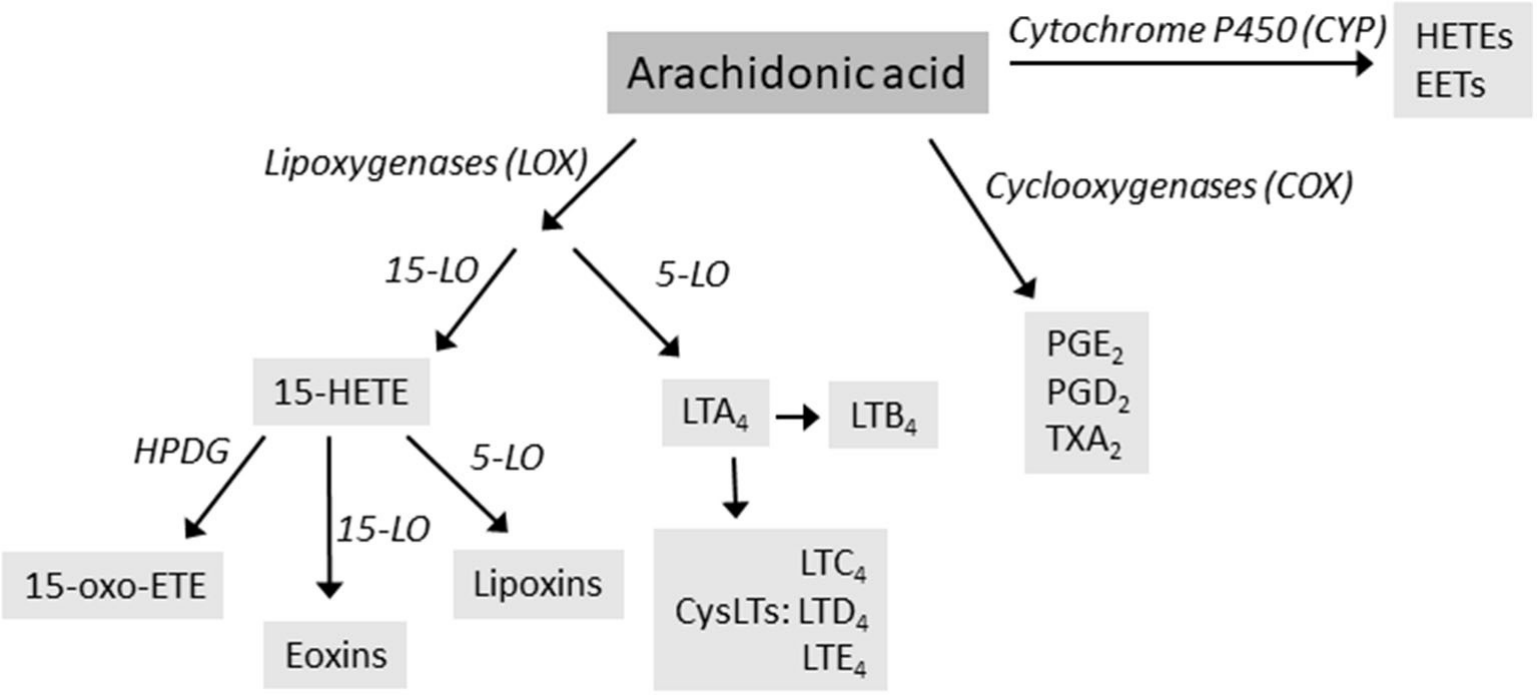
The main routes of arachidonic acid metabolism, focusing on lipoxygenase (LOX) pathway. Enzymes are in italics. HETE, hydroxyeicosatetraneoic acid; LT, leukotrienes; PG, prostaglandins; TX, thromboxanes.

Arachidonic acid (AA) is a C20 polyunsaturated fatty acid derived from phospholipid hydrolysis at the inner surface of the cell membrane by phospholipase PLA_2_. AA regulates phospholipase and protein kinase signaling as a second messenger, and acts as a key inflammatory intermediate, being a precursor for eicosanoids. Eicosanoids, which encompass prostaglandins (PGs), prostacyclins, thromboxanes (TXs) and leukotrienes (LTs), are hormone-like compounds signaling via G-coupled receptors and are involved in variety of biological processes, such as inflammation, platelet aggregation, electrolyte balance, and smooth-muscle contraction (4). Leukotrienes are synthesized via lipoxygenase (LOX)-dependent route, while PGs, prostacyclins and TXs are derived from the cyclooxygenase (COX)-dependent pathway (Figure 1). AA can also be metabolized by cytochrome P450 (CYP) enzyme, resulting in hydroxyeicosatetraneoic acids (HETEs) and epoxyeicosatrienoic acids (EETs), important in cardiovascular biology (4). AA metabolism is a complex entity, resulting in variety of lipid mediators that can be anti-inflammatory, proinflammatory, or both, depending on the target receptors.

### Pathophysiologic Findings of Arachidonic Acid Metabolism in AERD Patients

In AERD, AA metabolism is chronically imbalanced and both the COX- and the LOX-pathways are dysregulated. There is overproduction of CysLTs, increase in the expression of CysLT receptors in respiratory mucosa and in the LTC_4_ synthase enzyme, decrease in the lipoxin levels, decrease in prostaglandin PGE_2_ and increase in PGD_2_ [reviewed in (5)]. 5-lipoxygenase (5-LO) has gained a lot of attention because of the 30-year-old finding that AERD patients have increased levels of the 5-LO pathway end-product CysLTs, as measured by high levels of the stable end-metabolite LTE_4_ in urine and nasal fluids (6). Inhibiting the COX1 enzyme with NSAIDs further increases these levels. LT antagonists, such as montelukast, inhibit the actions of CysLTs by blocking their receptor CysLT1R, and have been shown to improve AERD-patients' asthma (7) and in some patients also inhibit the lower airway symptoms induced by NSAID ingestion (8).

### ATAD—Changes in Clinical Features

Another treatment option for non-responsive CRSwNP disease is aspirin treatment after desensitization (ATAD) (9). Successful desensitization has been shown to suppress the levels of PGD_2_, while the levels of CysLTs remain unaltered or even increase, and the levels of blood eosinophils and basophils increase (2). PGD_2_ induces chemotaxis in Th2 cells, eosinophils, and basophils (10), and the increased levels of circulating eosinophils might be due to their decreased tissue recruitment (2). A prospective, placebo-controlled, double-blind study comparing the effects of ATAD between ASA-intolerant and ASA-tolerant patients with asthma and CRSwNP (34 patients altogether) has shown improvement in the nasal and bronchial symptoms in the ASA-intolerant group (11). Clinical improvements were shown in SNOT-20 and Asthma Control Questionnaire scores, peak nasal inspiratory flows and as reduced doses of inhaled corticosteroids. In this study, the levels of LTE_4_ or PGD_2_ metabolites showed no changes after ATAD (11). It has been estimated, that over 80% of patients would benefit from ATAD (12) as it can reduce the need for surgeries, oral corticosteroids, and emergency room visits (13).

The downside of ATAD are the side effects: gastric pain or bleeding, bruising, tinnitus, urticaria or worsening of the respiratory symptoms, leading to discontinuation in 15% of cases (13, 14). Side effects may even prevent the desensitization, and acutely intolerable side effects during the aspirin desensitization procedure have been associated with elevated levels of PGD_2_ (2). A recent real-life study in the Finnish population reported ATAD-discontinuation rates of nearly 30%, in spite of lower ASA dose than the average described in literature (15). Another study from Finland reported similar results with a discontinuation rate of 22% (16), suggesting that there might be ethnic or genetic variability in ASA tolerance.

### 15-LO

Another enzyme that metabolizes AA is 15-lipoxygenase (15-LO), encoded by *ALOX-15* and expressed in airway epithelial cells, eosinophils and mast cells (17). It converts AA to 15-HETE, lipoxins and eoxins. 15-HETE can act as an independent anti-inflammatory mediator, or it can be converted to lipoxins by 5-LO, or to 15-oxo-eicosatetraeonic (15-oxo-ETE) by hydroxyprostaglandin dehydrogenase (HPGD) (18). However, epithelial cells lack HPGD and instead the enzyme is found in tissue mast cells in close proximity to the epithelial cells. The cells work transmetabolically, passing on the 15-HETE from the epithelial cell to the mast cell, which in turn produces 15-oxo-ETE (19). The role of 15-oxo-ETE in AERD progression is not known but it may contribute to the dysregulation of AA metabolism.

Increased levels of 15-HETE have been associated with pulmonary eosinophilia in asthmatics (20). Eoxins are proinflammatory metabolites capable of causing severe asthma and allergic reactions. They are produced by eosinophils and mast cells within the nasal polyp tissue (21). 15-LO activity was found to be increased in eosinophils isolated from asthmatics with either severe disease or AERD, and the levels of eoxins were specifically increased in asthmatics with AERD (22). *ALOX-15* is also upregulated in the epithelium in other type 2 inflammatory mucosal diseases such as eosinophilic esophagitis (23). Microarray experiments in human peripheral monocytes showed that the expression of 15-LO is strongly induced by interleukins (IL)-4 and IL-13, and real-time PCR indicated that IL-4 induced more than 100-fold upregulation of 15-LO expression (24).

Recently, a genome-wide association study (GWAS) of patients with CRSwNP across several cohorts showed that a missense variant of *ALOX-15*, causing a p.Thr560Met alteration and leading to near total loss of 15-LO enzymatic activity was associated with a reduction in the risk of CRSwNP (25). This was the first GWAS, and so far, the only one, to report a significant association with nasal polyps. Results from mouse models support this finding, as a mouse model deficient in 12/15-LO, an ortholog for human 15-LO, has been shown to have reduced airway inflammation and remodeling in allergen provocation tests (26).

Furthermore, a single-cell RNA sequencing analysis from nasal polyp tissue revealed dysregulated arachidonic metabolism in the 15-LO pathway in patients with AERD (19). When the polyp tissue isolated from CRSwNP patients was compared to that of AERD patients, *ALOX-15* expression was significantly elevated in patients with AERD, particularly within apical epithelial cells (19). Patients with CRSwNP and asthma had higher enhancement of whole-tissue *ALOX-15* expression compared to CRSwNP non-asthmatics. The same correlation was not observed in patients with non-polyp CRS. Patients with higher *ALOX-15* expression suffered from a worse CRSwNP disease with higher number of sinus operations and worse inflammation in the sinus CT scans (19). The expression level of *ALOX-15* mRNA was significantly higher in eosinophilic polyps and could distinguish between eosinophilic and non-eosinophilic nasal polyps (27). The expression of 15-LO was seen in both the epithelial cells and eosinophils in nasal polyp tissue detected by immunohistochemical staining (27). Dupilumab, a biological drug for treating asthma, atopic dermatitis and CRSwNP, suppresses IL-4 and IL-13 signaling, on which the expression of *ALOX-15* is strictly dependent (28). Dupilumab has been shown to be particularly therapeutically effective in AERD patients (29).

### Local Immunoglobulin Levels in AERD Nasal Polyps

Activation of B cells and local antibody production may play a key role in nasal polyp severity and AERD pathogenesis (30). The airway mucosa can function as a tertiary lymphoid organ where antibody production and class switching are facilitated as high levels of active B cells, plasma cells and plasmablasts have been identified locally (31, 32). IgE is a link between antibodies and inflammatory disease as it activates and can prime mast cells, basophils and other Fc-receptor bearing effector cells in nasal tissue (30). Other local antibodies may also promote inflammation. IgA enhances eosinophil survival (33) and IgG can activate local complement cascades leading to destruction of the epithelial barrier (34). One theory is that the presence of autoantibodies contribute to the destruction of this barrier, but no single antigen has consistently been linked to AERD.

Recently Buchheit et al. showed that the local antibody profile of patients with AERD differs from that of other CRSwNP patients, non-polyp CRS, and controls (35). In AERD, higher amounts of all subclasses of immunoglobulins were found, but especially of IgG4 and IgE. Local IgE levels did not correlate with serum IgE levels, indicating the presence of local antibody production within the tissues (35). Also, *IGHG4* encoding the constant region of IgG4 is overexpressed locally in AERD patients, strengthening the theory of local antibody production (35). Further, local IgE levels are associated with a worse disease and fast regrowth of polyposis whereas IgG4 associates with lifetime disease duration of AERD (35). It is speculated that IgG4 might prevent polyp regrowth, possibly causing a fibrotic disease in the sinuses (36, 37) but IgG4 might also be a step in the class switching into local IgE as *IL10* encoding IL10 cytokine that drives immunoglobulin production toward IgG4 has been shown in RNA-sequencing analysis of nasal polyp cells of AERD patients (35).

IgE antibody production has also been proposed to be driven by superantigens derived from bacteria such as *Staphylococcus aureus*, as enterotoxin-specific antibodies that may play a role in the inflammation cascade have been identified (38). A role for IL-5 has also been suspected in this eosinophilic disease, as IL-5 is an eosinophil activator that stimulates IgA production, produced by Th2 cells and mast cells. In AERD patients, antibody-expressing cells expressed *IL5AR* encoding for the IL-5 alfa receptor (35). This finding supports a possible additional pathway toward the presence of increased antibody levels in nasal polyps.

The positive effect of the IgE-binding monoclonal antibody omalizumab on CRSwNP showed in randomized, placebo-controlled studies POLYP1 and POLYP2 further strengthens the evidence of IgE playing part in the pathomechanism of AERD (39). In these identical studies that included 265 patients, 27% had AERD. After 24 weeks of treatment with omalizumab, polyp score decreased by 2 points or more in 31% of the patients in the active treatment group. Significant improvement compared to placebo was shown in all primary and secondary end points, i.e., nasal congestion score, SNOT-22, olfactory test UPSIT, and total nasal symptom score (39).

### Cells Involved in AERD and Their Interactions

**Figure 2:**
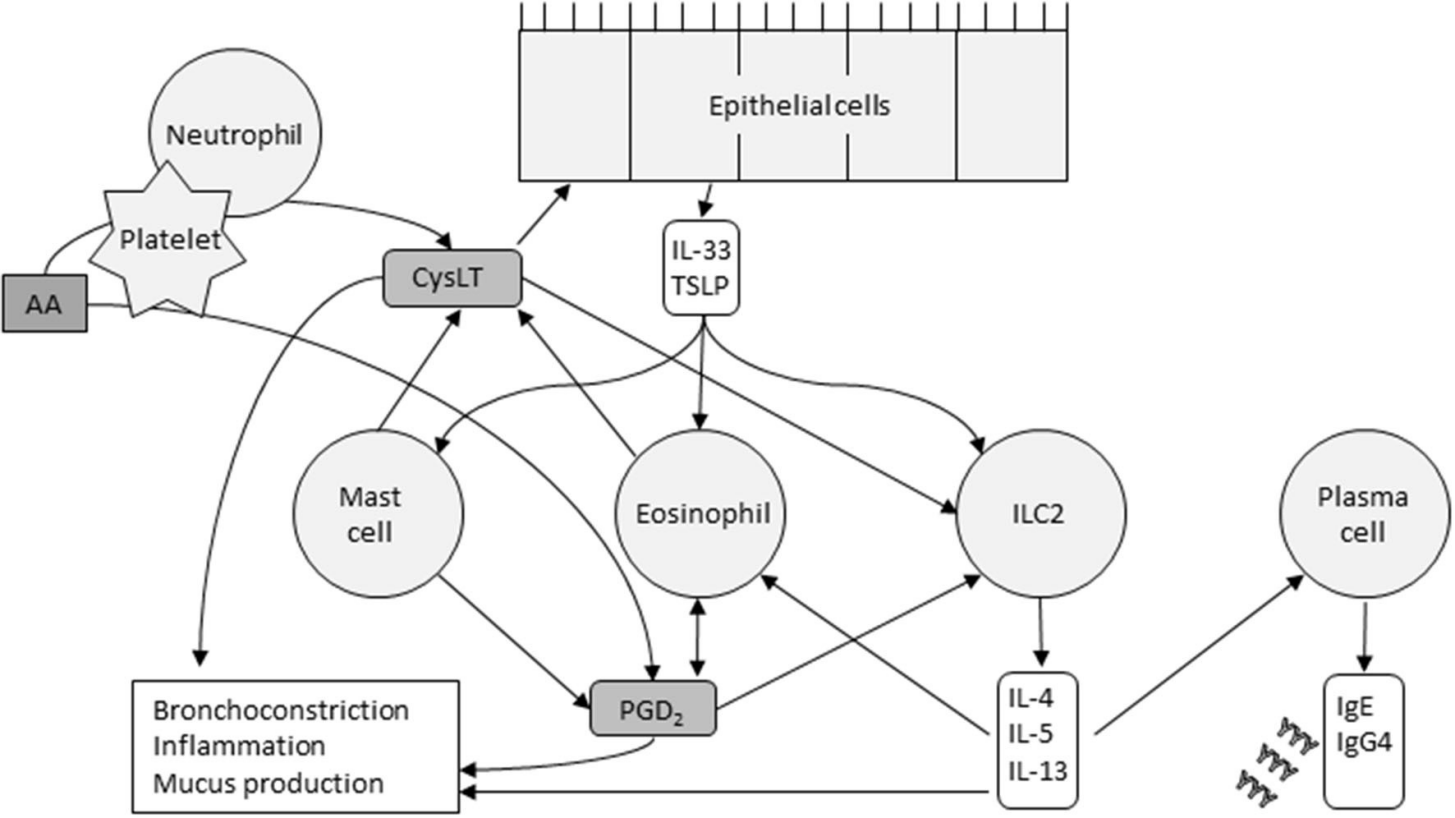
Cells and their major interactions in AERD. CysLTs released by mast cells, eosinophils, and platelets cause epithelial release of IL-33 and TSLP. These innate cytokines in turn activate mast cells, eosinophils, and ILC2s. The cytokines released by ILC2s, the pathways of which are now targeted by several monoclonal antibodies, stimulate plasma cells to produce IgE and IgG4, and promote, recruit and activate eosinophils. Eosinophils and ICL2s are also activated by PGD_2_. Platelets metabolize arachidonic acid (AA) into PGD_2_, and, in collaboration with neutrophils, CysLTs. The inflammatory response is driven by multiple factors.

AERD is characterized by an imbalance in eicosanoid levels, especially CysLTs, PGD_2_ and PGE_2_. Interactions between the cells that produce eicosanoids and the cells that respond to them play a key role in the AERD pathomechanism. Mast cells, eosinophils, epithelial cells, platelets, and innate lymphoid cell type 2 (ILC2) cells are thought to be the major players involved in these actions (Figure 2).

Eosinophils, abundant in nasal polyps, bronchial mucosa, and peripheral blood, produce PGD_2_ and are attracted to PGD_2_ by chemotaxis (40). Choi et al. found that eosinophils interact with epithelial cells through surfactant protein D, and may mediate smooth muscle remodeling, a clinical feature of AERD (41). Eosinophils are activated by many effectors including PGD_2_, IL-5, IL-3, IL-33 and thymic stromal lymphopoietin (TSLP). Upon activation, they release CysLTs and other mediators that promote type 2 inflammatory reactions and tissue damage (42).

Mast cells and their activation through CysLT is central in AERD-related inflammation and aspirin-induced reactions. Mast cells can be activated through IgE cross-linking or via the innate alarmin IL-33. Recently, Pan et al. demonstrated that in mouse models, COX-1 activity is required for IL-33-dependent mast cell release of AA (43), a mechanism that may explain aspirin desensitization. IL-33 and the innate alarmin TSLP induce mast cells' production of PGD_2_ (3) and potentiate each other's actions. PGD_2_ promotes bronchospasm and inflammatory cell recruitment, which is typical in AERD but mast cells also release inflammatory mediators such as histamine and tryptase, and CysLTs.

CysLTs released by mast cells and eosinophils regulate the release of IL-33 and TSLP from the basal epithelial cell layer of the airways. In addition to activating mast cells, TSLP and IL-33 also activate eosinophils and ILC2 cells. These cells are a rare subset of lymphocytes and upon stimulation, release cytokines such as IL-4, IL-5, IL-13, and IL-9. The three first mentioned may stimulate plasma cells to produce IgE and IgG4. IL-5 also promotes recruitment, survival and activation of eosinophils and IL-9 may increase the amount of mast cells recruited to the tissue (40). Several prostaglandins also regulate ILC2 function. PGD_2_ activates ILC2s and induces chemotaxis and cytokine production, whereas PGE_2_ and PGI_2_ have inhibitory actions (40). During aspirin desensitization, ILC2s in nasal fluid increased in correlation with symptom scores and with increases in PGD_2_ metabolites in urine, indicating a potential connection between PGD_2_ release, ILC2 recruitment, and symptom severity during aspirin-induced reactions in AERD (44).

Platelets and neutrophils also work transmetabolically in AERD (45). Activated platelets in AERD express high surface levels of P-selectin (46) which acts as an adhesion molecule (47). Platelets adhere to neutrophils, cells that generate LTA_4_. Neutrophils, however, lack LTC_4_ synthase, which is needed to metabolize LTA_4_ further into LTC_4_ (45). The adherent platelets express LTC_4_ synthase, and the neutrophil-platelet aggregates can then function together to allow for CysLT overproduction.

### Lipid Dysregulation in AERD

Because the fatty acid metabolism is dysregulated in AERD, dietary modifications that could balance the distortion and decrease the symptoms have been investigated. The dietary intake of AA and its precursor, linoleic acid, correlates to the amount of AA in inflammatory cells, while supplementation with omega-3-rich fatty acids has been shown to decrease the bodies' production of inflammatory leukotrienes (48). A 2-week diet high in omega-3 and low in omega-6 fatty acids significantly decreased the urinary end-metabolites of LTE_4_ and PGD_2_, as well as the clinical end-points of upper respiratory symptom control (SNOT-22 score) and asthma control (ACQ score) (49). Low-salicylate diet has been evaluated in a trial of 30 patients, showing benefits for both subjective and objective clinical end-points (50). However, the effects of non-acetylated salicylates on AERD end-metabolites have not been investigated and the mechanism by which dietary salicylates would affect the respiratory symptoms is not clear.

A totally new finding of macrophage memory and their epigenetic reprogramming in AERD was recently reported by Haimerl et al. (51), assessed by RNA sequencing, metabolomics flux assays and LC-MS/MS. Together with reported dysregulation in sphingolipid metabolism (52), they report increased release of acylcarnitine metabolites, indicating a broader dysregulation in fatty acid metabolism in AERD. Haimerl et al. also reported that although the expression profile of macrophages showed upregulation of proinflammatory genes, it also showed reduction on host-protective molecules. They speculated that a dysfunctional macrophage activation state may contribute to defect in pathogen clearance and higher risk of exacerbations in AERD patients (51).

## Discussion

AERD is characterized by chronic inflammation with an imbalance in eicosanoids, high levels of inflammatory mediators such as CysLTs and PGD_2_, and mast cell, platelet, and ILC2 activation. Upon activation, ILC2s release interleukins that can be blocked with new monoclonal antibodies that are used in the treatment of severe asthma and CRSwNP (39, 53, 54). The inflammation type in asthma and CRSwNP is mostly type 2, but significant heterogeneity in the inflammatory background in AERD patients has been reported (55). The more we learn about the pathomechanisms of these inflammatory diseases, and especially about the subset AERD, the more we can personalize treatment.

In the future, systems biology methods could give us more insight on the cellular level changes in this disease. Already now, the pathways behind CRS and asthma have been investigated with different omics approaches (genomics, transcriptomics, proteomics and metabolomics) [reviewed in (56, 57)]. The findings have made biomarker analysis and point-of-care technology development realistic, thus allowing more precise and quicker diagnoses and personalized treatment to be available in the future clinical practice.

## Author Contributions

AL and ML contributed to conception and design of the study and wrote the first draft of the manuscript. All authors contributed to the article and approved the submitted version.

## Conflict of Interest

The authors declare that the research was conducted in the absence of any commercial or financial relationships that could be construed as a potential conflict of interest.

## Publisher's Note

All claims expressed in this article are solely those of the authors and do not necessarily represent those of their affiliated organizations, or those of the publisher, the editors and the reviewers. Any product that may be evaluated in this article, or claim that may be made by its manufacturer, is not guaranteed or endorsed by the publisher.
